# A Case of Primary Mediastinal Seminoma Associated With Testicular Microlithiasis and Liver Injury

**DOI:** 10.7759/cureus.14527

**Published:** 2021-04-16

**Authors:** Ana Colon Ramos, Kidist Tarekegn, Camelia Ciobanu, Harry G Sequeira Gross, Ivette Vigoda

**Affiliations:** 1 Internal Medicine, St. Barnabas Hospital, Bronx, USA; 2 Oncology, Albert Einstein Medical Center, St. Barnabas Healthcare System, Bronx, USA

**Keywords:** mediastinal seminoma, extragonadal germ cell tumor, testicular microlithiasis, liver injury, congestive hepatopathy

## Abstract

Mediastinal seminomas are rare neoplasms that can be found incidentally in asymptomatic patients. However, a few cases may present in the emergency room with mild to severe respiratory and/or cardiovascular symptoms. This can occur when the tumor grows large enough to cause compression and obstruction of the various structures present in the thorax. Here we present a case of a large medium mediastinal seminoma that grew to the extent of causing pulmonary artery compression which led to chronic passive backflow through the hepatic veins and hepatic congestion. This case was remarkable as well for the presence of testicular microlithiasis, a rare feature with unknown significance to date.

## Introduction

The extragonadal germ cell tumor (EGGCT) corresponds to a germ cell neoplasm localized anywhere except in the gonads. It may appear near almost every structure along the midline of the body from the brain to the coccyx, most notably in the anterior mediastinum, central nervous system (CNS), and retroperitoneum. It represents 1% to 3% of all GCTs and can be classified as seminomatous, which comprises 60% of all primary EGGCT formed only by the classical seminoma, and non-seminomatous including embryonal carcinoma (EC), teratoma (mature or immature), yolk sac carcinoma (YST) and choriocarcinoma. EGGCT constituted by two or more histotypes is referred to as mixed germ cell tumor [1,2].

The EGGCT comprises only 15% of the anterior mediastinal masses where they are frequently found incidentally on imaging studies in asymptomatic, usually male patients aged 20 to 40 years. The size of the mass can range from 1 to 20 cm. In advanced cases, a CT of the chest may show a large mass with sharply demarcated borders, often with homogeneous attenuation. Biochemical markers such as B-hCG are frequently elevated. Histologically, cells are large, have a regular nucleus with a prominent nucleolus, and have a distinct cell membrane exhibiting abundant clear eosinophilic cytoplasm containing glycogen. A lymphoid infiltrate constituted by small mature lymphocytes is commonly present [3-8].

A thorough physical examination is necessary during the initial evaluation of these patients, paying special attention to the gonads to screen for any coexisting GCT using imaging tools as simple as a testicular ultrasound. In the case of a primary EGGCT, this ultrasound will be negative, or on rare occasions, it may show microlithiasis which consists of multiple, randomly distributed, echogenic micronodules with no acoustic shadow, measuring 1-3 mm in diameter. A testicular microlithiasis (TM) combined with a mediastinal germ cell tumor is a rare finding that has been reported in no more than 8 cases [9-16].

Although the mediastinal seminoma usually remains contained in the chest, it has the ability to become very large and infiltrate the surrounding organs at early stages. If it grows large enough, it may cause cardiac and/or respiratory compromise manifested as difficulty in breathing secondary to obstruction of the bronchial and vascular structures which can lead to serious complications, including but not limited to pneumonia and right-sided heart failure [17]. Any pathology capable of causing right-sided heart failure can result in congestive hepatopathy which is usually painless and may be suggested only by abnormal liver biochemistry during a routine evaluation. Here we present a case of EGGCT in the mediastinum associated with TM and liver injury.

## Case presentation

A 32-year-old male presented to the emergency room with chief complaints of shortness of breath on exertion associated with dry cough and atypical chest pain that worsened when lying down. The review of systems was positive for weight loss, night sweats and fatigue for approximately three months. He denied fever, hemoptysis, recent travel or sick contacts. Medical history is remarkable only for malaria. He works as a housekeeper at a restaurant, is a light smoker, drinks alcohol occasionally and denies any recreational drug use. On the physical exam, he had tachycardia at rest and decreased breath sounds throughout the left lung. There was no palpable cervical, supraclavicular or axillary adenopathies. Laboratory blood tests were remarkable for moderate anemia with iron studies suggesting an anemia of chronic disease, thrombocytosis, elevated transaminases and high alkaline phosphatase (Table 1). The liver biochemical tests were persistently abnormal prior to any therapeutic intervention (Table 2).

**Table 1:** Pertinent blood count, metabolic panel and iron studies. RBC: red blood cell; Hgb: hemoglobin; WBC: white blood cell; AST: aspartate aminotransferase; ALT: alanine aminotransferase; ALP: alkaline phosphatase; INR: international normalized ratio; TIBC: total iron-binding capacity.

Variable	On admission	Reference range
RBC	4.02	4.63-6.08x10^6^/uL
Hgb	9	13.7-17.5 gm/dL
WBC	4.8	4.2-9.1x10^3^/uL
Platelet count	485	163-337x10^3^/uL
AST	56	8-33 IU/L
ALT	77	4-36 IU/L
ALP	205	38-126 IU/L
Total bilirubin	0.7	0.1-1.2 mg/dL
Albumin	3	3.8-5 gm/dL
INR	1.3	0.9-1.1 Ratio
Total iron	20	65-175 ug/dL
TIBC	173.6	250-400 ug/dL
Iron saturation	11.5	20-55%
Ferritin	978	15-200 ng/ml

**Table 2:** Liver biochemical markers AST: aspartate aminotransferase; ALT: alanine aminotransferase; ALP: alkaline phosphatase.

Variable	Hospital day 1	Hospital day 2	Hospital day 35
AST IU/L	56	120	62
ALT IU/L	77	125	84
ALP IU/L	205	195	321

**Figure 1:**
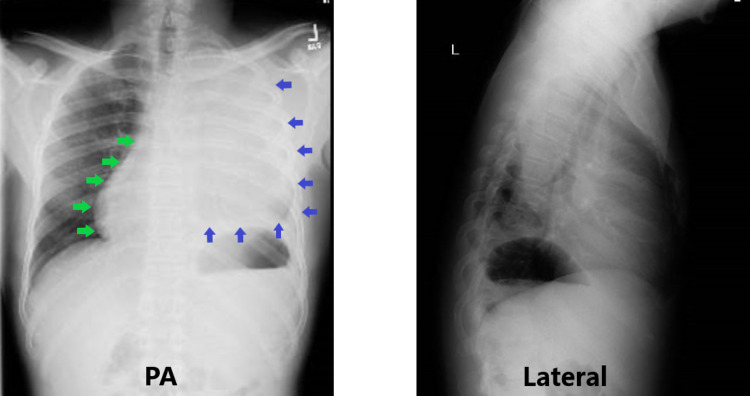
Posteroanterior (PA) and lateral chest x-ray. Complete opacification of the left hemithorax (blue arrows). Rightward cardiomediastinal shift (green arrows).

**Figure 2:**
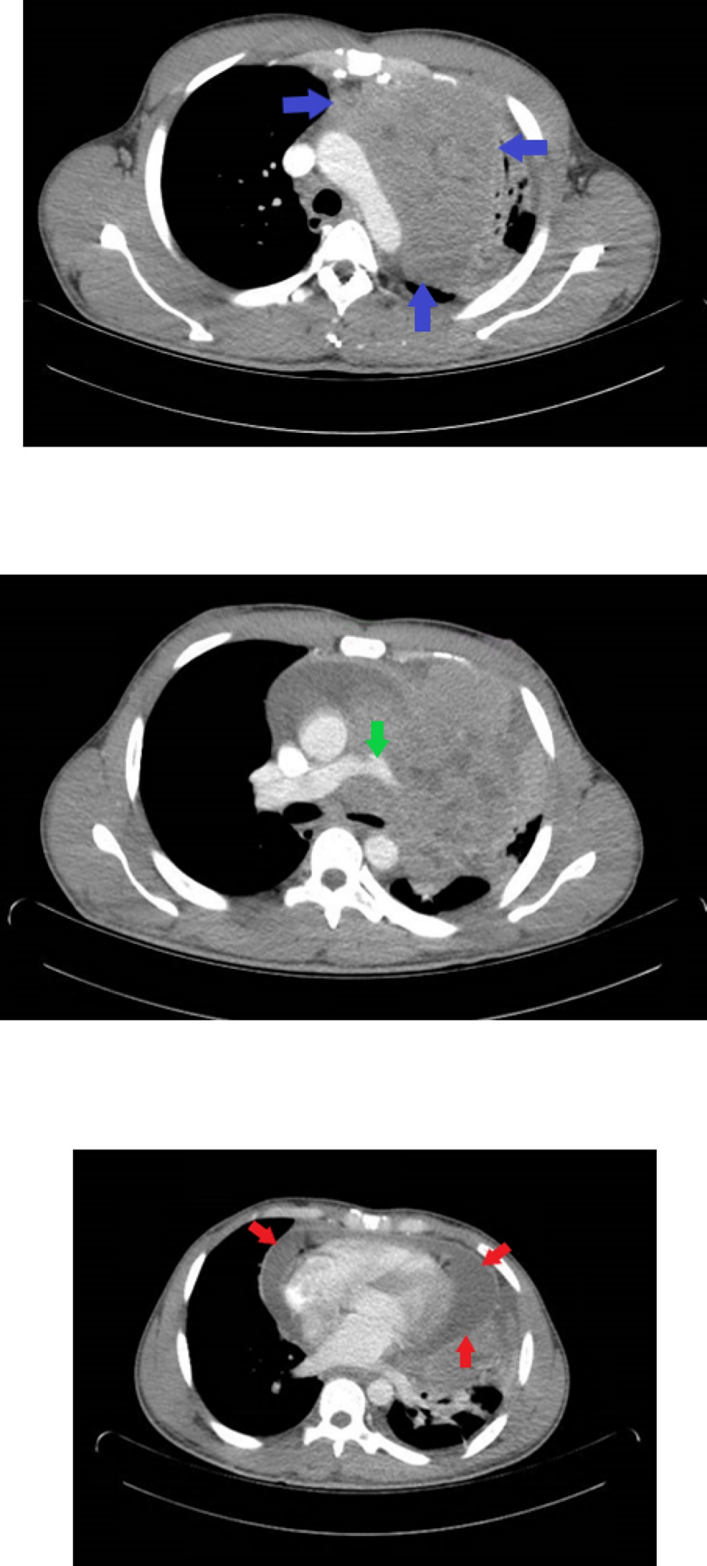
Contrast-enhanced CT of the chest. Left thoracic mass measuring 14.1 cm longitudinally, nearly 13 cm anteroposteriorly and nearly 13 cm transversely with regions of inner necrosis arising from the middle mediastinum (top image, blue arrows). Severe narrowing of the left pulmonary artery (middle image, green arrow). Moderate pericardial effusion is noted as well (lower image, red arrows)

**Figure 3:**
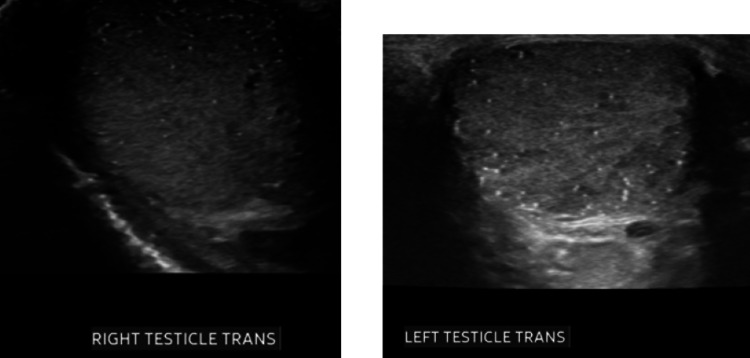
Testicular ultrasound. Right and left testicles showing echogenic small dots representing intratubular calcifications better known as testicular microlithiasis.

**Figure 4:**
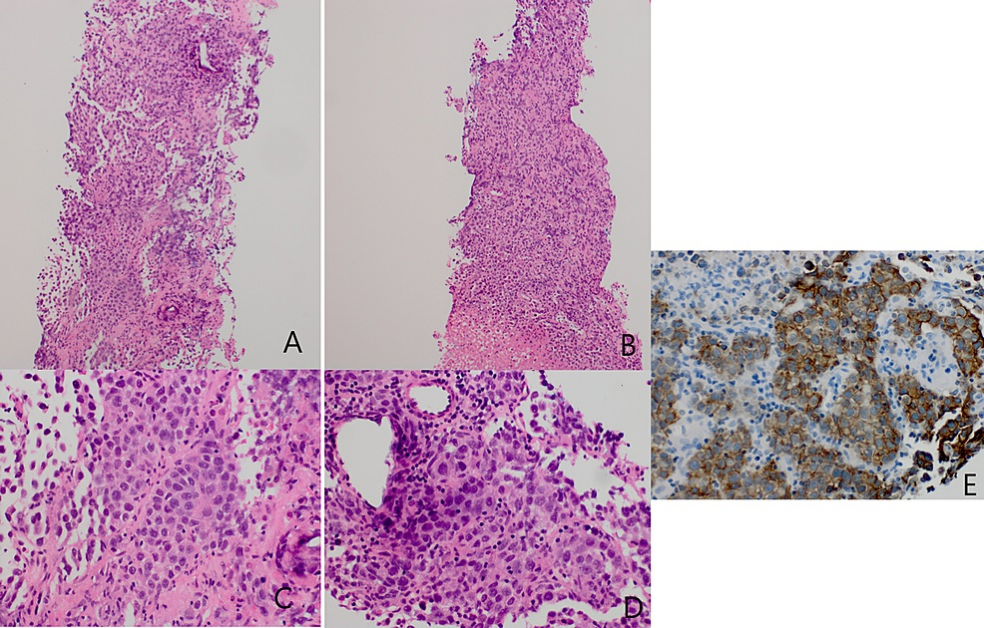
Pathology of the biopsied lesion. (A & B): H&E stained slides show nests of neoplastic cells with intermixed small lymphocytes and extensive necrosis. (original magnification 100x); (C & D): at higher power, the neoplastic cells have pinpoint-to-focally conspicuous nucleoli and focal apoptosis. (original magnification 400x ); (E): the neoplastic cells stain with c-kit (E), SALL4, OCT4 and D2-40 (data not shown) (original magnification 400x).

Blood cultures, influenza test, legionella antigen in the urine and acid-fast bacillus (AFB) stain in the sputum were negative. The chest X-ray showed complete opacification of the L side of the lung as well as rightward cardiomediastinal shift (Figure 1). EKG showed voltage criteria for LVH and T wave inversion from V3-V6. A transthoracic echocardiogram showed mild tricuspid valve regurgitation, a right ventricular systolic pressure of 27 mmHg, small-mod pericardial effusion and a large L pleural effusion. A liver ultrasound was performed to evaluate the acute liver injury and ALP elevation but no abnormalities in the liver parenchyma were seen. A contrast CT chest, abdomen and pelvis showed a large L hemithoracic mass. There was also extrinsic compression of the left mainstem bronchus and encasement with severe narrowing of the left pulmonary artery. Rightward cardiomediastinal shift, invasion of the left side of the pericardium and pericardial effusion was also noted (Figure 2). There was no evidence of skeletal metastases. Hepatomegaly and elevated L hemidiaphragm were also seen (not shown in the figures). The evidence of a mediastinal solid mass along with the clinical presentation raised concerns for a metastatic vs primary neoplastic mass. Further workup included: alpha-fetoprotein (AFP): 1.4 ng/ml (normal range: 0.0-8.3 ng/ml), Lactate dehydrogenase (LDH): 464 IU/L (normal range: 100-190 IU/L) and beta-human chorionic gonadotropin tests (B-HCG): 43 mIU/L (normal range: 0-5 mIU/L). An MRI of the brain didn’t show any metastasis. Physical examination of the testicles was normal. A bilateral scrotal ultrasound demonstrated bilateral microlithiasis and small bilateral varicoceles. No masses were identified (Figure 3). A CT-guided biopsy of the mediastinal mass reported a pathology consistent with a germ cell tumor, seminoma type (Figure 4).

Treatment outcome

The patient was on the third cycle (C3) of bleomycin, etoposide and platinum (BEP) at the time of the publication. The C1 was complicated by grade 1 neutropenia requiring neupogen for three days. The liver biochemical tests pre- and post-chemotherapy are presented in the following chart (Table 3).

**Table 3:** Effect of treatment of the tumor on the liver enzymes. AST: aspartate aminotransferase; ALT: alanine aminotransferase; ALP: alkaline phosphatase.

Pre-treatment				Post-treatment			
AST (IU/L)	56	120	62	25	28	20	15
ALT (IU/L)	77	125	84	44	41	22	13
ALP (IU/L)	205	195	321	230	188	128	79

**Figure 5:**
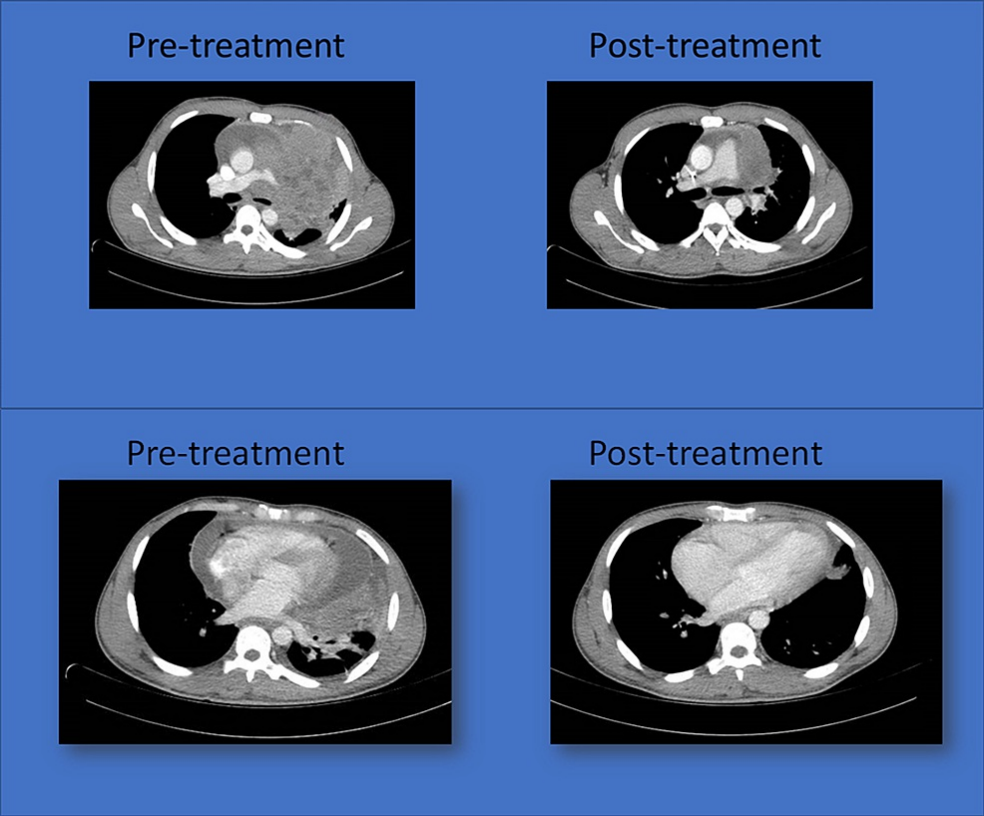
Comparison of the pre-treatment and post-treatment contrast-enhanced CT scan of the chest. Evidence of the decrease in the size of the mass (images on the top) and resolution of the pericardial effusion (images on the bottom).

A re-staging CT performed after C2 showed decreased size of the left anterior mediastinal mass now measuring 9.8 x 4.7 x 6.3 cm encasing the left main pulmonary artery and appears to be a nonocclusive chronic emboli present in the left main pulmonary artery. The mass presented with less invasion of the mediastinum compared to the prior examination. There is a marked improvement in the aeration of the left lung since the mass has decreased in size and less obliteration of the left mainstem bronchus (Figure 5). Areas of atelectasis have improved in the left upper lobe and lingula. Mild areas of scarring of the left lower lobe are noted. After completion of C3 he will get re-staging with a CT scan. If the disease is resolved, no further therapy will be needed. 

## Discussion

The seminomatous mediastinal germ cell tumor is a rare pathology that has been reported over 100 times already. Its association with bilateral TM is an extremely rare finding with an unknown significance in this setting. This is the 9th case report of a mediastinal EGGCT associated with testicular microlithiasis. Its clinical utility it’s not yet proven but there are theories suggesting this finding as a premalignant lesion [15]. If the presence of testicular microlithiasis plays a role in the natural history of the disease or as an indicator of genetic susceptibility to develop later in life an EGGCT, it remains to us as unsolved questions. The combination of testicular microlithiasis in a patient with cardiopulmonary signs and/or symptoms such as cough, dyspnea, chest pain or jugular venous distention (JVD) should raise the concern of an EGGCT of the mediastinum. In addition, a chest radiograph could be considered in selective cases. However, to date, there’s no consensus for screening patients with TM for any EGGCT as this finding needs to be better characterized.

Congestive hepatopathy is usually asymptomatic and can result from any cause of right-sided heart failure such as mitral stenosis, tricuspid regurgitation, constrictive pericarditis, cor pulmonale and cardiomyopathy. In this case report, the patient had a CT of the chest showing compression of the L main bronchi and pulmonary artery which could explain his cardiopulmonary symptoms. An echocardiography showed mild tricuspid regurgitation which could be provoked by the back flow of the blood from the lungs decreasing the venous return causing the congestion of the liver parenchyma and subsequent hepatomegaly. This explains the elevation of the hepatic enzymes, a sign of liver injury. It wasn't determined whether the elevated alkaline phosphatase (ALP) was of liver origin due to intrahepatic ductal dilatation secondary to passive backflow throughout the liver or not. Studies have shown that the serum alkaline phosphatase level may be elevated in chronic heart failure along with elevation of aminotransferase levels in about one third of the patients but typically no more than 2x to 3x the upper limit of normal [18,19]. The imaging tests didn’t show any signs of obstruction or lesions in the biliary tree and liver parenchyma respectively that could offer an alternative explanation to this finding. However, gamma glutamyltransferase (GGT), which is usually absent in placental tissue, was never measured to make this distinction. Another strong possibility for the elevated ALP might be the production of placental alkaline phosphatase by the seminoma itself as reported in many cases of seminomatous tumor [20]. The cardiopulmonary symptoms as well as the liver biochemical tests abnormalities were resolved as soon as the chemotherapy was started, likely due to the decrease in the size of the tumor and relief of the cardiovascular and respiratory obstruction.

## Conclusions

A mediastinal germ cell tumor presenting with features of liver injury is a unique scenario. Although the signs of liver injury raise a concern of primary hepatic disease, the features suggestive of congestive hepatopathy that resolved after the initiation of the chemotherapy confirmed that the liver injury was a secondary phenomenon. Clinicians should continue reporting the presence of testicular microlithiasis in patients with EGGCT as well as the associated features in order to grant a definitive meaning in particular if this is a premalignant lesion. This case report highlights the heterogeneous presentations of germ cell tumors and the importance of carefully considered workup to reach the correct conclusion.
